# Synthesis and properties of oligonucleotides modified with an *N*-methylguanidine-bridged nucleic acid (GuNA[Me]) bearing adenine, guanine, or 5-methylcytosine nucleobases

**DOI:** 10.3762/bjoc.17.54

**Published:** 2021-03-04

**Authors:** Naohiro Horie, Takao Yamaguchi, Shinji Kumagai, Satoshi Obika

**Affiliations:** 1 Graduate School of Pharmaceutical Sciences, Osaka University, 1-6 Yamadaoka, Suita, Osaka 565-0871, Japan; 2 Sohyaku. Innovative Research Division, Mitsubishi Tanabe Pharma Corporation, Shonan Health Innovation Park, 2-26-1 Muraoka-Higashi, Fujisawa, Kanagawa 251-8555, Japan; 3 National Institutes of Biomedical Innovation, Health and Nutrition (NIBIOHN), 7-6-8 Saito-Asagi, Ibaraki, Osaka 567-0085, Japan

**Keywords:** artificial nucleic acid, duplex-forming ability, oligonucleotide synthesis

## Abstract

Chemical modifications have been extensively used for therapeutic oligonucleotides because they strongly enhance the stability against nucleases, binding affinity to the targets, and efficacy. We previously reported that oligonucleotides modified with an *N*-methylguanidine-bridged nucleic acid (GuNA[Me]) bearing the thymine (T) nucleobase show excellent biophysical properties for applications in antisense technology. In this paper, we describe the synthesis of GuNA[Me] phosphoramidites bearing other typical nucleobases including adenine (A), guanine (G), and 5-methylcytosine (^m^C). The phosphoramidites were successfully incorporated into oligonucleotides following the method previously developed for the GuNA[Me]-T-modified oligonucleotides. The binding affinity of the oligonucleotides modified with GuNA[Me]-A, -G, or -^m^C toward the complementary single-stranded DNAs or RNAs was systematically evaluated. All of the GuNA[Me]-modified oligonucleotides were found to have a strong affinity for RNAs. These data indicate that GuNA[Me] could be a useful modification for therapeutic antisense oligonucleotides.

## Introduction

**Figure 1:**
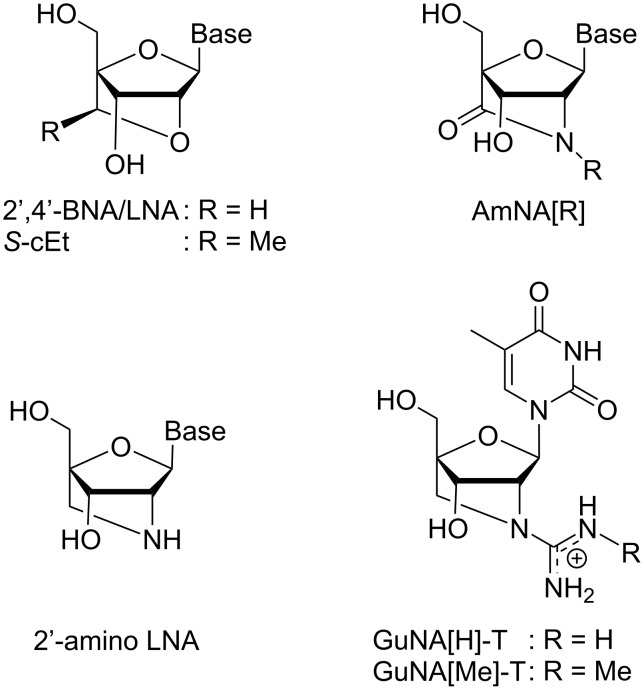
Structures of 2',4'-BNA/LNA analogs.

The efficacy and safety of therapeutic oligonucleotides can be controlled by chemical modifications. For applications in antisense technology, chemical modifications aimed at enhancing the duplex-forming ability toward a target RNA (i.e., a complementary single-stranded RNA) and improving the stability against enzymatic degradations are commonly utilized. For instance, antisense oligonucleotides (ASOs) modified with 2',4'-bridged nucleic acid/locked nucleic acid (2',4'-BNA/LNA; Figure 1) are now widely used for gene regulation in vitro and in vivo because 2',4'-BNA/LNA greatly increases the affinity toward the target RNAs, thus enhancing the efficacy of the modified ASOs [1–6]. Notably, the biophysical and pharmacological properties of 2',4'-BNA/LNA-modified ASOs can be further altered with subtle structural changes. Seth and co-workers developed the *S*-2',4'-constrained-2'-*O*-ethyl (*S*-cEt; Figure 1) derivative, which has an exocyclic methyl group in its bridged structure [7–8]. The *S*-cEt-modified ASOs displayed a higher nuclease resistance and lower hepatotoxicity in in vivo experiments than the corresponding 2',4'-BNA/LNA-modified ASOs [9]; the reduction in hepatotoxicity might be a sequence-dependent phenomenon. Currently, a number of *S*-cEt-modified ASOs with low hepatotoxicity have been confirmed to be effective for gene regulations in vivo [10–11]. We previously developed amido-bridged nucleic acids (AmNA[R]s) (Figure 1), in which the *N*-alkyl substituent groups were found to modulate nuclease resistance and hepatic distributions [12]. Wengel’s group reported the synthesis of 2'-amino-LNA (Figure 1) functionalized with a peptide or sugar at the N2'-position, with the aim of modulating the physicochemical properties and specific organ distributions of the therapeutic oligonucleotides [13–14]. A more favorable example is the covalent attachment of a guanidine moiety, which is a common approach to partially neutralize the polyanionic property of oligonucleotides [15–18]. In our previous study, a guanidine-bridged nucleic acid (GuNA[H]; Figure 1) bearing a thymine (T) nucleobase was synthesized as a novel artificial nucleic acid for antisense applications [19]. The modification of oligonucleotides with GuNA[H]-T improved the nuclease resistance, cell membrane permeability, and binding affinity toward complementary single-stranded DNAs (ssDNAs) and RNAs (ssRNAs). We also synthesized and evaluated a GuNA[H]-T analog bearing a methyl group in the guanidine moiety (GuNA[Me]-T; Figure 1) [20]. The GuNA[Me]-T exhibited a similar duplex-forming ability and nuclease resistance as GuNA[H]-T. Since a subtle change in the structure of the 2',4'-BNA/LNA modulated its biophysical and pharmacological properties, in vivo experiments with GuNA[H] and GuNA[Me] are expected to provide further mechanistic insights into how small substituents affect the efficacy and safety of therapeutic oligonucleotides. Thus, the synthesis of GuNA[Me] phosphoramidites bearing other typical nucleobases, i.e., adenine (A), guanine (G), or 5-methylcytosine (^m^C), instead of the immunologically unfavorable cytosine (C), is needed.

The preparation of all four phosphoramidites (A, G, ^m^C, and T) is generally not easy because each nucleobase differs in the sensitivity to reactions, and appropriate protecting groups need to be selected [8,21–23]. We recently achieved the synthesis of all four GuNA[H] phosphoramidites, where transglycosylations of the 2'-amino-LNA analog with the corresponding nucleobases were performed as the key reactions [24–25]. The transglycosylation is a powerful strategy that simplifies the preparation of phosphoramidites at the late stages of the syntheses [26–27]. Here, we describe the synthesis of GuNA[Me]-A, -G, and -^m^C phosphoramidites and their incorporations into oligonucleotides. The duplex-forming abilities of all the GuNA[Me]-modified oligonucleotides toward their ssDNA and ssRNA complements were systematically evaluated.

## Results and Discussion

### Synthesis of the GuNA[Me] phosphoramidites bearing either an A, G, or ^m^C nucleobase

**Scheme 1:**
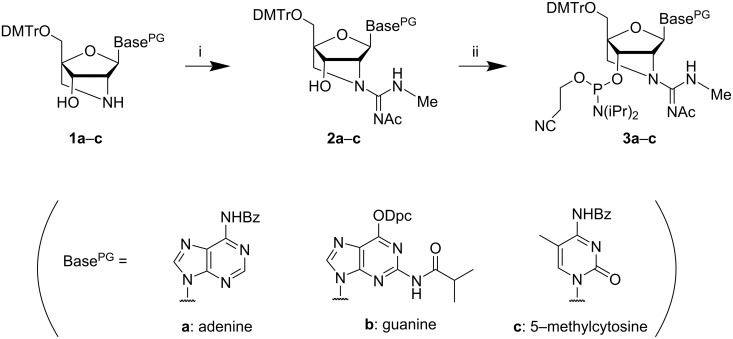
The preparation of the GuNA[Me]-A, -G, and -^m^C phosphoramidites **3a**–**c**. Reagents and conditions: (i) *N*-acetyl-*S*,*N'*-dimethylisothiourea, AgOTf, DIPEA, THF, rt, 72% (**2a**), 65% (**2b**), 83% (**2c**); (ii) (iPr)_2_NP(Cl)O(CH_2_)_2_CN, DIPEA, CH_2_Cl_2_, rt, 87% (**3a**); 65% (**3b**); 72% (**3c**).

The preparation of the GuNA[Me]-A, -G, and -^m^C phosphoramidites **3a**–**c** needed for the synthesis of the GuNA[Me]-modified oligonucleotides is detailed in Scheme 1. The acetyl group was selected as a protecting group for the guanidine moiety because it can be easily removed under the basic conditions (ammonia/methylamine solution) used for the DNA synthesis [20]. The phosphoramidite synthesis was started from 2'-amino-LNAs **1a**–**c**, which were rapidly prepared via the transglycosylations of 2'-amino-LNA-T [25]. First, the 2'-amino groups of **1a**–**c** were converted into guanidine moieties with a methyl group using *N*-acetyl-*S*,*N'*-dimethylisothiourea [28], which yielded 65–83% of the products **2a**–**c**. Subsequently, the designed GuNA[Me] phosphoramidites **3a**–**c** were successfully obtained following the phosphitylation of the 3'-hydroxy groups of **2a**–**c**. Notably, since the nucleobases were introduced at the late stage of the synthesis, we had no difficulty preparing these phosphoramidites.

### Synthesis of oligonucleotides modified with GuNA[Me]-A, -G, or -^m^C

The prepared GuNA[Me]-A, -G, and -^m^C phosphoramidites were incorporated into the middle position of 12-mer oligonucleotides (Table 1). The oligonucleotide synthesis was performed using an automated DNA synthesizer following the established synthetic method for GuNA[Me]-T-modified oligonucleotides [20]. 5-(Ethylthio)-1*H*-tetrazole (ETT) was used as an activator for the coupling, and the coupling time was extended from 40 s to 20 min for the GuNA[Me] phosphoramidites. Other conditions were the same as those used for general DNA synthesis. After the elongation, the oligonucleotides were treated with ammonia/methylamine solution (7 M NH_3_ in methanol/40% aqueous methylamine 1:1) at 60 °C for 5 h. Under these conditions, we obtained the GuNA[Me]-^m^C-modified oligonucleotide **ON3** with high purity. In the case of the GuNA[Me] having a purine nucleobase (**ON1** and **ON2**), the acetyl group in the guanidine moiety remained in a considerable amount. This means that we should give attention to the reactivity of each nucleobase. Finally, the acetyl group was successfully removed by extending the deprotection time to 10 h. The yield range of the designed oligonucleotides **ON1**–**ON3** was 12–25%, as shown in Table 1.

**Table 1:** Synthetic yields and mass spectral data of the GuNA[Me]-modified oligonucleotides **ON1**–**ON3**.

oligonucleotides^a^ (5'–3')		yield [%]	MALDI–TOF mass	
	
			found [M − H]^−^	calcd. [M − H]^−^

d(GCG TTA TTT GCT)	(**ON1**)	12	3723.9	3724.5
d(GCG TTG TTT GCT)	(**ON2**)	14	3738.9	3740.5
d(GCG TT^m^C TTT GCT)	(**ON3**)	25	3714.4	3714.5

^a^A, G, and ^m^C indicate GuNA[Me] modifications.

### Duplex-forming ability of oligonucleotides modified with GuNA[Me]-A, -G, or -^m^C

The binding affinity of the GuNA[Me]-modified oligonucleotides **ON1**–**ON3** toward ssDNAs or ssRNAs was evaluated by measuring UV melting temperatures (*T*_m_ values), and the obtained values were compared with those of the corresponding unmodified oligonucleotides (**ON6**–**ON8**). The results are shown in Table 2. As expected, all of the GuNA[Me]-modified oligonucleotides **ON1**–**ON3** exhibited markedly higher *T*_m_ values toward ssRNAs than their unmodified counterparts **ON6**–**ON8** (Δ*T*_m_ = 5–6 °C). These results are similar to those obtained for the GuNA[Me]-T-modified oligonucleotide **ON4** (Δ*T*_m_ = 5 °C). Additionally, the modified **ON1**–**ON3** showed an enhanced duplex-forming ability toward the complementary ssDNAs (Δ*T*_m_ = 3–6 °C). Among them, GuNA[Me]-A-modified **ON1** exhibited a slightly lower Δ*T*_m_ value than others. This type of nucleobase-dependent difference in Δ*T*_m_ values is also seen in other GuNA[H]-modified oligonucleotides [25]. Since oligonucleotides modified with 2',4'-BNA/LNA or its analog scpBNA show different nucleobase dependency [1,23], these results could be considered characteristic of the GuNA-modified oligonucleotides. Interactions between the guanidine moiety and nearby base pairing(s) might have affected the Δ*T*_m_ values, though further investigations are needed for the details.

**Table 2:** *T*_m_ values of duplexes formed between GuNA[Me]-modified oligonucleotides and complementary ssRNAs or ssDNAs.^a^

oligonucleotides^a^ (5'–3')		*T*_m_ (Δ*T*_m_) [°C]			
	
		vs ssRNA		vs ssDNA	

d(GCG TTT TTT GCT)^b^	(**ON5**)	47		51	
d(GCG TTT TTT GCT)^b^	(**ON4**)	52	(+5)	56	(+5)

d(GCG TTA TTT GCT)	(**ON6**)	45		49	
d(GCG TTA TTT GCT)	(**ON1**)	50	(+5)	52	(+3)

d(GCG TTG TTT GCT)	(**ON7**)	51		54	
d(GCG TTG TTT GCT)	(**ON2**)	57	(+6)	59	(+5)

d(GCG TTC TTT GCT)	(**ON8**)	52		53	
d(GCG TT^m^C TTT GCT)	(**ON3**)	58	(+6)	59	(+6)

^a^Conditions: 10 mM sodium phosphate buffer (pH 7.2), 100 mM NaCl, 4 µM each oligonucleotide, 0.5 °C/min at 260 nm. Sequences of the complementary ssRNA and ssDNA are 5'-r(AGC AAA NAA CGC)-3' and 5'-d(AGC AAA NAA CGC)-3', respectively. T, A, G, and ^m^C indicate GuNA[Me] modifications. ^b^See reference [20].

### CD spectral analyses of duplexes modified with GuNA[Me]-G

**Figure 2:**
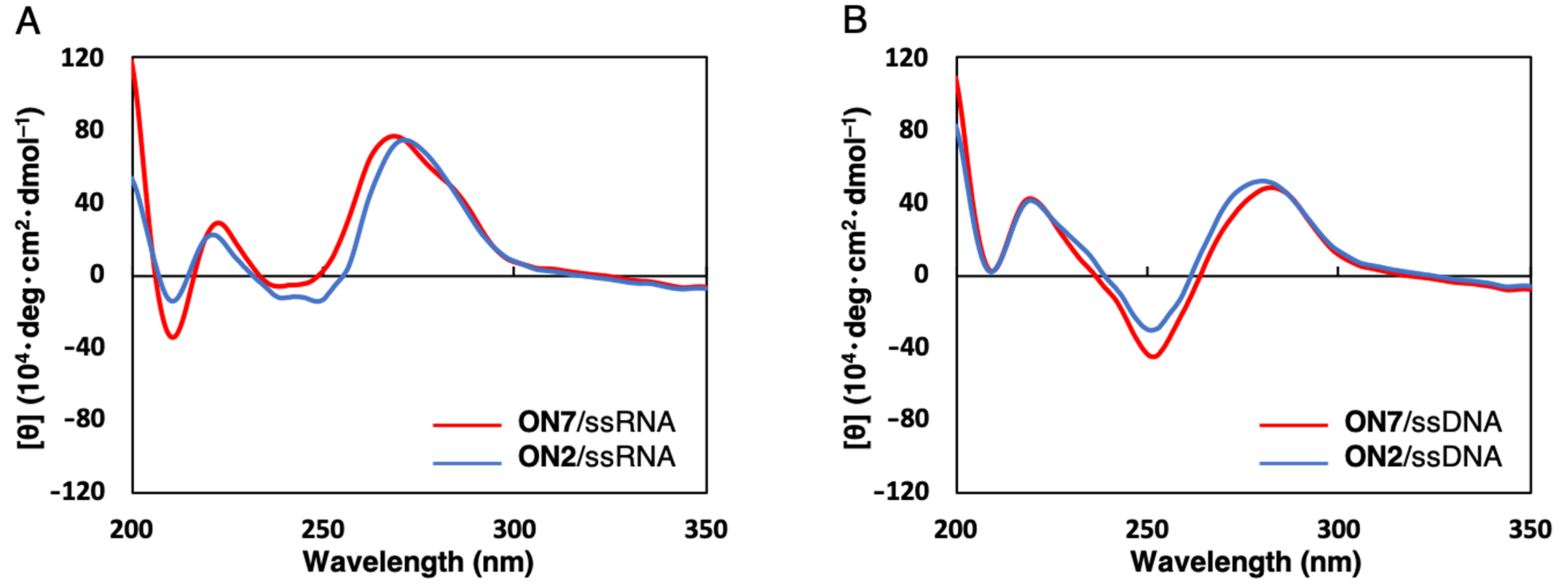
The CD spectra of the **ON7**/ssRNA, **ON2**/ssRNA, **ON7**/ssDNA, and **ON2**/ssDNA duplexes. Conditions: 10 mM sodium phosphate buffer (pH 7.2), 100 mM NaCl, 4 µM each oligonucleotide. Sequences of the complementary ssRNA and ssDNA are 5'-r(AGC AAA CAA CGC)-3' and 5'-d(AGC AAA CAA CGC)-3', respectively.

To analyze the structures of the duplexes containing GuNA[Me], circular dichroism (CD) spectra were measured for **ON2**/ssRNA and **ON2**/ssDNA duplexes (Figure 2). The CD spectra of **ON2**/ssRNA and **ON2**/ssDNA were found to be similar to those of the **ON7**/ssRNA and **ON7**/ssDNA duplexes, demonstrating that one modification with GuNA[Me] does not affect the whole duplex structures. Similar results were observed for **ON4**/ssRNA and **ON4**/ssDNA (Figure S15 in Supporting Information File 1). In our previous studies, DNA/RNA (A-form) duplexes containing a multiple GuNA[H] modification displayed similar spectral patterns to the natural and the 2',4'-BNA/LNA-modified counterparts [19]. Since GuNA[Me] showed similar results to GuNA[H] in terms of the duplex-forming ability [25], a multiple GuNA[Me] modification to A-form duplexes is also believed not to affect the structures.

## Conclusion

We successfully synthesized GuNA[Me] phosphoramidites bearing either an A, G, or ^m^C nucleobase. Each monomer was derived from the corresponding 2'-amino-LNA in two steps and introduced into oligonucleotides. By protecting the guanidine moieties with an acetyl group, we could obtain the oligonucleotides within a 12–25% yield range under the basic conditions (ammonia/methylamine solution) commonly used in oligonucleotide synthesis. The synthesized GuNA[Me]-modified oligonucleotides showed a high binding affinity toward the complementary ssRNAs and ssDNAs. Considering the facile synthesis of the GuNA[Me] monomers and the ability of the GuNA[Me]-modified oligonucleotides to form stable duplexes with ssRNAs, we expect that a modification using GuNA[Me] could be useful for antisense applications. In our ongoing studies, we are evaluating the efficacy of ASOs modified with GuNAs in vitro and in vivo, and the results will be reported in due course.

## Experimental

### Chemicals and instrumentation

All moisture-sensitive reactions were carried out in well-dried glassware under N_2_ atmosphere. Dehydrated acetonitrile, dichloromethane, and tetrahydrofuran (THF) were used as purchased. ^1^H, ^13^C, and ^31^P NMR spectra were recorded using a JEOL JNM-ECS300 spectrometer. The chemical shift values are expressed in δ values (ppm) relative to tetramethylsilane as an internal standard, CHCl_3_ (δ = 7.26 ppm) for ^1^H NMR, CDCl_3_ (δ = 77.0 ppm) for ^13^C NMR, and 5% H_3_PO_4_ (δ = 0 ppm) for ^31^P NMR. Infrared (IR) spectra were recorded using a JASCO FT/IR-4200 spectrometer. The optical rotation was recorded using a JASCO P-2200 instrument. A MALDI–TOF mass spectrometer (SpiralTOF JMS-S3000) was used to measure the mass spectra of all compounds. For column chromatography, silica gel PSQ 60B or 100B was used. The progress of the reactions was monitored by analytical thin-layer chromatography on glass plates (TLC Silica gel 60 F_254_), and the products were visualized using UV light.

### Synthesis of phosphoramidites

**(1*R*,3*R*,4*R*,7*S*)-5-(*N'*-Acetyl-*N*-methylcarbamimidoyl)-3-(*N*^6^-benzoyladenine-9-yl)-1-(4,4'-dimethoxytrityl)oxymethyl-2-oxa-5-azabicyclo[2.2.1]heptan-7-ol (2a):** This compound was synthesized in a similar manner as described in reference [20]. To the mixture of compound **1a** (841 mg, 1.23 mmol) and *N*-acetyl-*S*,*N'*-dimethylisothiourea (271 mg, 1.84 mmol), anhydrous THF (12 mL) was added and the mixture placed in an ice bath under stirring. Subsequently, *N*,*N*-diisopropylethylamine (0.35 mL, 2.0 mmol) and silver triflate (507 mg, 1.97 mmol) were added, and the mixture was stirred at room temperature overnight. Upon completion of the reaction, the mixture was diluted with ethyl acetate, after which sat. aq. NaCl was added. Following filtration, the product was extracted with ethyl acetate, washed with water and brine, dried (using Na_2_SO_4_), and concentrated. The product was then purified using column chromatography to yield **2a** (698 mg, 72%) as a yellow solid substance. **2a**: 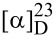 −26.4 (*c* 1.0, CHCl_3_); IR (KBr): 2999, 2952, 2837, 1696, 1606, 1509, 1451, 1410, 1297, 1251, 1177, 1155, 1074, 1035 cm^−1^; ^1^H NMR (CDCl_3_) δ 2.00 (s, 3H), 2.80 (s, 3H), 3.48, 3.57 (AB, *J* = 10.7 Hz, 2H), 3.67 (s, 2H), 3.74 (s, 3H), 3.74 (s, 3H), 4.37 (s, 1H), 5.02 (s, 1H), 6.14 (s1H), 6.79 ( d, *J* = 8.9 Hz, 4H), 7.15–7.33 (m, 7H), 7.42–7.61 (m, 5H), 7.98 (dd, *J* = 1.4 Hz, 7.2 Hz, 2H), 8.25 (s, 1H), 8.70 (s, 1H), 9.15 (s, 1H); ^13^C NMR (CDCl_3_) δ 26.44, 29.70, 54.23, 55.16, 60.27, 63.88, 71.06, 85.51, 86.46, 88.50, 113.18, 123.47, 126.95, 127.86, 127.90, 128.03, 128.84, 129.94, 130.01, 132.87, 133.31, 135.21, 135.47, 140.48, 144.26, 149.38, 150.80, 152.55, 158.52, 164.76; HRMS–MALDI (*m*/*z*): [M + Na]^+^ calcd for C_43_H_42_N_8_O_7_Na, 805.3069; found, 805.3063.

**(1*R*,3*R*,4*R*,7*S*)-5-(*N'*-Acetyl-*N*-methylcarbamimidoyl)-1-(4,4'-dimethoxytrityl)oxymethyl-3-(*O*^6^-diphenylcarbamoyl*-N*^2^-isobutyrylguanine-9-yl)-2-oxa-5-azabicyclo[2.2.1]heptan-7-ol (2b):** This compound was synthesized in a similar manner as described in reference [20]. To the mixture of compound **1b** (2.15 g, 2.49 mmol) and *N*-acetyl-*S,N'*-dimethylisothiourea (402 mg, 2.75 mmol), anhydrous THF (25 mL) was added and the mixture placed in an ice bath under stirring. Subsequently, *N*,*N*-diisopropylethylamine (0.57 mL, 3.3 mmol) and silver triflate (835 mg, 3.25 mmol) were added, and the mixture was stirred at room temperature for 2 h. Upon completion of the reaction, the mixture was diluted with ethyl acetate and washed with sat. aq. NaHCO_3_, after which sat. aq. NH_4_Cl was added. Following filtration, the product was extracted with ethyl acetate, washed with water and brine, dried (using Na_2_SO_4_), and concentrated. The product was purified using column chromatography to yield **2b** (1.56 g, 65%) as a yellow solid substance. **2b**: 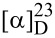 −15.2 (*c* 1.0, CHCl_3_); IR (KBr): 3350, 2971, 2837, 1750, 1712, 1587, 1509, 1444, 1411, 1335, 1284, 1249, 1226, 1176, 1116, 1068, 1035 cm^−1^; ^1^H NMR (CDCl_3_) δ 1.23 (d, *J* = 6.5 Hz, 3H), 1.25 (d, *J* = 6.2 Hz, 3H), 2.05 (s, 3H), 2.55–2.66 (m, 1H), 3.05 (d, *J* = 4.2 Hz, 3H), 3.48, 3.53 (AB, *J* = 10.8 Hz, 2H), 3.59, 3.74 (AB, *J* = 10.3 Hz, 2H), 3.78 (s, 3H), 3.78 (s, 3H), 4.29 (s, 1H), 5.07 (s, 1H), 6.00 (s, 1H), 6.83 (d, *J* = 7.9 Hz, 4H), 7.16–7.45 (m, 18H), 8.13 (s, 1H), 8.16 (s, 1H); ^13^C NMR (CDCl_3_) δ 19.19, 19.34, 26.03, 29.96, 36.61, 54.14, 55.14, 60.15, 63.64, 72.12, 85.49, 86.35, 88.17, 113.19, 121.57, 126.91, 127.90, 128.02, 129.18, 129.94, 129.99, 135.31, 135.52, 141.55, 144.34, 150.26, 151.60, 153.12, 156.07, 158.50, 162.61, 174.91; HRMS (MALDI) (*m*/*z*): [M + Na]^+^ calcd. for C_53_H_53_N_9_O_9_Na, 982.3858; found, 982.3856.

**(1*R*,3*R*,4*R*,7*S*)-5-(*N'*-Acetyl-*N*-methylcarbamimidoyl)-1-(4,4'-dimethoxytrityl)oxymethyl-3-(*O*^6^-diphenylcarbamoyl*-N*^2^-isobutyrylguanine-9-yl)-2-oxa-5-azabicyclo[2.2.1]heptan-7-ol (2c):** This compound was synthesized in a similar manner as described in reference [20]. To the mixture of compound **1c** (679 mg, 1.01 mmol) and *N*-acetyl-*S,N'*-dimethylisothiourea (194 mg, 1.33 mmol), anhydrous THF (10 mL) was added and the mixture placed in an ice bath under stirring. Subsequently, *N*,*N*-diisopropylethylamine (0.28 mL, 1.6 mmol) and silver triflate (411 mg, 1.60 mmol) were added, and the mixture was stirred at room temperature for 1 h. Upon completion of the reaction, the mixture was diluted with ethyl acetate, after which sat. aq. NH_4_Cl was added. Following filtration, the product was extracted with ethyl acetate, washed with water and brine, dried (using Na_2_SO_4_), and concentrated. The product was purified using column chromatography to yield **2c** (641 mg, 83%) as a white solid substance. **2c**: ^1^H NMR (CDCl_3_) δ 1.82 (s, 3H), 2.03 (d, *J* = 4.2 Hz, 3H), 2.81 (s, 3H), 3.31, 3.51 (AB, *J* = 9.7 Hz, 2H), 3.48, 3.57 (AB, *J* = 10.9 Hz, 2H), 3.76 (s, 3H), 3.77 (s, 3H), 4.31 (s, 1H), 4.60 (s, 1H), 5.56 (s, 1H), 6.83 (dd, *J* = 8.6 Hz, 1.7 Hz, 4H), 7.21–7.55 (m, 12H), 7.72 (s, 1H), 8.30 (d, *J* = 7.2 Hz, 2H); ^13^C NMR (CDCl_3_) δ 13.62, 26.30, 29.47, 53.81, 54.04, 55.17, 58.97, 63.18, 69.93, 86.08, 86.66, 88.70, 111.89, 113.24, 127.03, 128.00, 128.07, 129.86, 129.94, 130.11, 132.52, 135.14, 135.57, 136.87, 144.23, 147.92, 158.59, 159.66, 161.16, 179.48; HRMS–MALDI (*m*/*z*): [M + Na]^+^ calcd for C_43_H_44_N_6_O_8_Na, 795.3113; found, 795.3106.

**(1*R*,3*R*,4*R*,7*S*)-5-(*N'*-Acetyl-*N*-methylcarbamimidoyl)-3-(*N*^6^-benzoyladenine-9-yl)-7-[2-cyanoethoxy(diisopropylamino)phosphanyl]oxyl-1-(4,4'-dimethoxytrityl)oxymethyl-2-oxa-5-azabicyclo[2.2.1]heptane (3a):** This compound was synthesized in a similar manner as described in reference [20]. To a solution of **2a** (1.47 g, 1.9 mmol) in dichloromethane (19 mL), *N*,*N*-diisopropylethylamine (0.7 mL, 4.1 mmol) and 2-cyanoethyl-*N*,*N*-diisopropylchlorophosphoramidite (0.8 mL, 3.8 mmol) were added, and the mixture was stirred at room temperature for 6 h. Upon completion of the reaction, sat. aq. NaHCO_3_ was added, and the product was extracted with dichloromethane. The organic phase was washed with water and brine, dried (using Na_2_SO_4_), and concentrated. The product was purified using column chromatography to yield **3a** (1.62 g, 87%) as a yellow solid substance. **3a**: ^31^P NMR (CDCl_3_) δ 149.15, 149.31; HRMS–MALDI (*m*/*z*): [M + Na]^+^ calcd for C_52_H_59_N_10_O_8_NaP, 1005.4147; found, 1005.4143.

**(1*R*,3*R*,4*R*,7*S*)-5-(*N'*-Acetyl-*N*-methylcarbamimidoyl)-7-[2-cyanoethoxy(diisopropylamino)phosphanyl]oxyl-1-(4,4'-dimethoxytrityl)oxymethyl-3-(*O*^6^-diphenylcarbamoyl*-N*^2^-isobutyrylguanine-9-yl)-2-oxa-5-azabicyclo[2.2.1]heptane (3b):** This compound was synthesized in a similar manner as described in reference [20]. To a solution of **2b** (149 mg, 0.155 mmol) in dichloromethane (1.5 mL), *N*,*N*-diisopropylethylamine (56 µL, 0.32 mmol) and 2-cyanoethyl-*N*,*N*-diisopropylchlorophosphoramidite (69 µL, 0.31 mmol) were added, and the mixture was stirred at room temperature for 6 h. Upon completion of the reaction, sat. aq. NaHCO_3_ was added, and the product was extracted with dichloromethane. The organic phase was washed with water and brine, dried (using Na_2_SO_4_), and concentrated. The product was purified using column chromatography to yield **3b** (117 mg, 65%) as a yellow solid substance. **3b**: ^31^P NMR (CDCl_3_) δ 148.80, 149.55; HRMS–MALDI (*m*/*z*): [M + Na]^+^ calcd for C_62_H_70_N_11_O_10_NaP, 1182.4937; found, 1182.4955.

**(1*R*,3*R*,4*R*,7*S*)-5-(*N'*-Acetyl-*N*-methylcarbamimidoyl)-7-[2-cyanoethoxy(diisopropylamino)phosphanyl]oxyl-1-(4,4'-dimethoxytrityl)oxymethyl-3-(*O*^6^-diphenylcarbamoyl*-N*^2^-isobutyrylguanine-9-yl)-2-oxa-5-azabicyclo[2.2.1]heptane (3c):** This compound was synthesized in a similar manner as described in reference [20]. To a solution of **2c** (1.08 g, 1.40 mmol) in dichloromethane (14 mL), *N*,*N*-diisopropylethylamine (0.8 mL, 4.3 mmol) and 2-cyanoethyl-*N*,*N*-diisopropylchlorophosphoramidite (0.6 mL, 2.8 mmol) were added, and the mixture was stirred at room temperature for 6 h. Upon completion of the reaction, sat. aq. NaHCO_3_ was added, and the product was extracted with dichloromethane. The organic phase was washed with water and brine, dried (using Na_2_SO_4_), and concentrated. The product was purified using column chromatography to yield **3c** (0.98 g, 72%) as a yellow solid substance. **3c**: ^31^P NMR (CDCl_3_) δ 148.61, 148.85; HRMS–MALDI (*m*/*z*): [M + Na]^+^ calcd for C_52_H_61_N_8_O_9_NaP, 995.4191; found, 995.4181.

### Oligonucleotide synthesis and purification

The synthesis of the oligonucleotides modified with GuNA[Me]-A, -G, or -^m^C (0.2 µmol scale) was performed using the nS-8 oligonucleotide synthesizer (GeneDesign, Inc.) according to the standard phosphoramidite protocol with 0.5 M 5-ethylthiotetrazole as an activator. The protocol is similar to that described in reference [20]. A Custom Primer Support™ T 40s (GE Healthcare) was used as a solid support. The amidite solution was dehydrated. The standard synthesis cycle was used for the assembly of the reagents except that the coupling time was extended to 16 min. The synthesis was carried out in the trityl-on mode. The oligonucleotides were treated with a 1:1 mixture of 7 N ammonia solution in methanol and 40% aq. methylamine at room temperature for 10 h to remove the solid support, and then the mixture was heated at 60 °C for 10 h (^m^C) or 15 h (A and G). After deprotection, the oligonucleotides were rapidly purified using a Sep-Pac^®^ Plus C18 Cartridge. Subsequently, the desired oligonucleotides were further purified using reversed-phase HPLC with Waters XBridge™ C18 (4.6 × 50 mm analytical and 10 mm × 50 mm preparative) columns, with a linear gradient of MeCN (2.5–5% over 5 min, then 5–7.5% over 20 min) in 0.1 M triethylammonium acetate buffer (pH 7.0). The purity and structure of the oligonucleotides were confirmed by HPLC and MALDI–TOF mass spectrometry, respectively.

### UV melting experiments and melting profiles

The UV melting experiments were carried out using SHIMADZU UV-1650PC and SHIMADZU UV-1800 spectrometers equipped with a *T*_m_ analysis accessory. Equimolecular amounts of the target ssRNAs or ssDNAs and the oligonucleotides were dissolved in 10 mM sodium phosphate buffer (pH 7.2) containing 100 mM NaCl to achieve a final strand concentration of 4 µM. The samples were annealed by heating at 95 °C followed by slow cooling to room temperature. The melting profile was recorded at 260 nm from 0 to 90 °C at a scan rate of 0.5 °C/min. The *T*_m_ values were taken as the temperatures at which the formed duplexes were half dissociated, determined by the midline of the melting curves.

### CD spectrum measurement

The CD spectra were recorded at 10 °C in a quartz cuvette of 1 cm optical path length. The samples were prepared in the same manner as described in the UV melting experiments. The molar ellipticity was calculated from the equation [θ] = θ/*cl*, where θ, *c*, and *l* indicate the relative intensity, sample concentration, and path length in centimeters, respectively.

## Supporting Information

File 1 ^1^H, ^13^C, and ^32^P NMR spectra for all new compounds, HPLC charts and MALDI–TOF mass data for all new oligonucleotides, UV melting curves of the duplexes formed between GuNA[Me]-modified oligonucleotides and ssDNAs (or ssRNAs), and CD spectra of **ON4**/ssRNA and **ON4**/ssDNA.
