# Supplementary material for: Synthesis and properties of oligonucleotides modified with an N-methylguanidine-bridged nucleic acid (GuNA[Me]) bearing adenine, guanine, or 5-methylcytosine nucleobases
Source: Beilstein J Org Chem. 2021 Mar 4;17:622–9. doi: 10.3762/bjoc.17.54 (PMC7940814; doi:10.3762/bjoc.17.54)
Supplement: File 1 — 1H, 13C, and 32P NMR spectra for all new compounds, HPLC charts and MALDI–TOF mass data for all new oligonucleotides, UV melting curves of the duplexes formed between GuNA[Me]-modified oligonucleotides and ssDNAs (or ssRNAs), and CD spectra of ON4/ssRNA and ON4/ssDNA. [file Beilstein_J_Org_Chem-17-622-s001.pdf]

BEILSTEIN JOURNAL OF ORGANIC CHEMISTRY

Supporting Information

for

Synthesis and properties of oligonucleotides modified with an *N*-methylguanidine-bridged nucleic acid (GuNA[Me]) bearing adenine, guanine, or 5-methylcytosine nucleobases

Naohiro Horie, Takao Yamaguchi, Shinji Kumagai and Satoshi Obika

Beilstein J. Org. Chem. **2021,** *17,* 622–629. doi:10.3762/bjoc.17.54

^{1}H, ^{13}C, and ^{32}P NMR spectra for all new compounds, HPLC charts and MALDI–TOF mass data for all new oligonucleotides, UV melting curves of the duplexes formed between GuNA[Me]-modified oligonucleotides and ssDNAs (or ssRNAs), and CD spectra of ON4/ssRNA and ON4/ssDNA

License and Terms: This is a supporting information file under the terms of the Creative Commons Attribution License (https://creativecommons.org/licenses/by/4.0). Please note that the reuse, redistribution and reproduction in particular requires that the author(s) and source are credited and that individual graphics may be subject to special legal provisions.
The license is subject to the *Beilstein Journal of Organic Chemistry* terms and conditions: (https://www.beilstein-journals.org/bjoc/terms)

Table of contents

1. ^{1}H NMR, ^{13}C NMR, and ^{31}P NMR spectra of new compounds

2. Characterisation of oligonucleotides

3. UV melting experiments

4. CD spectral analysis

1. ^{1}H NMR, ^{13}C NMR, and ^{31}P NMR spectra of new compounds

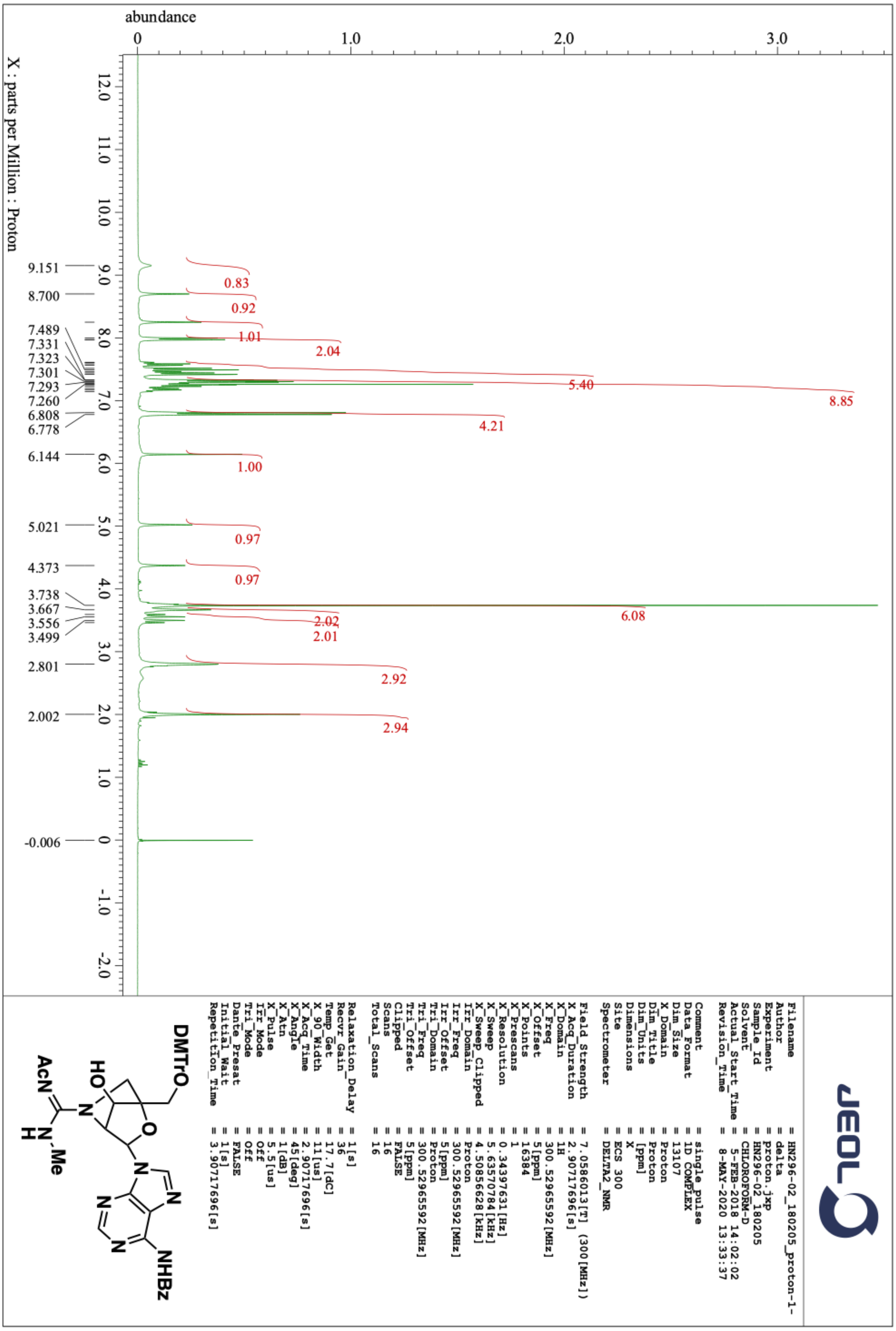

Figure S1: Compound **2a** (^{1}H NMR, $CDCl_3$, 300 MHz)

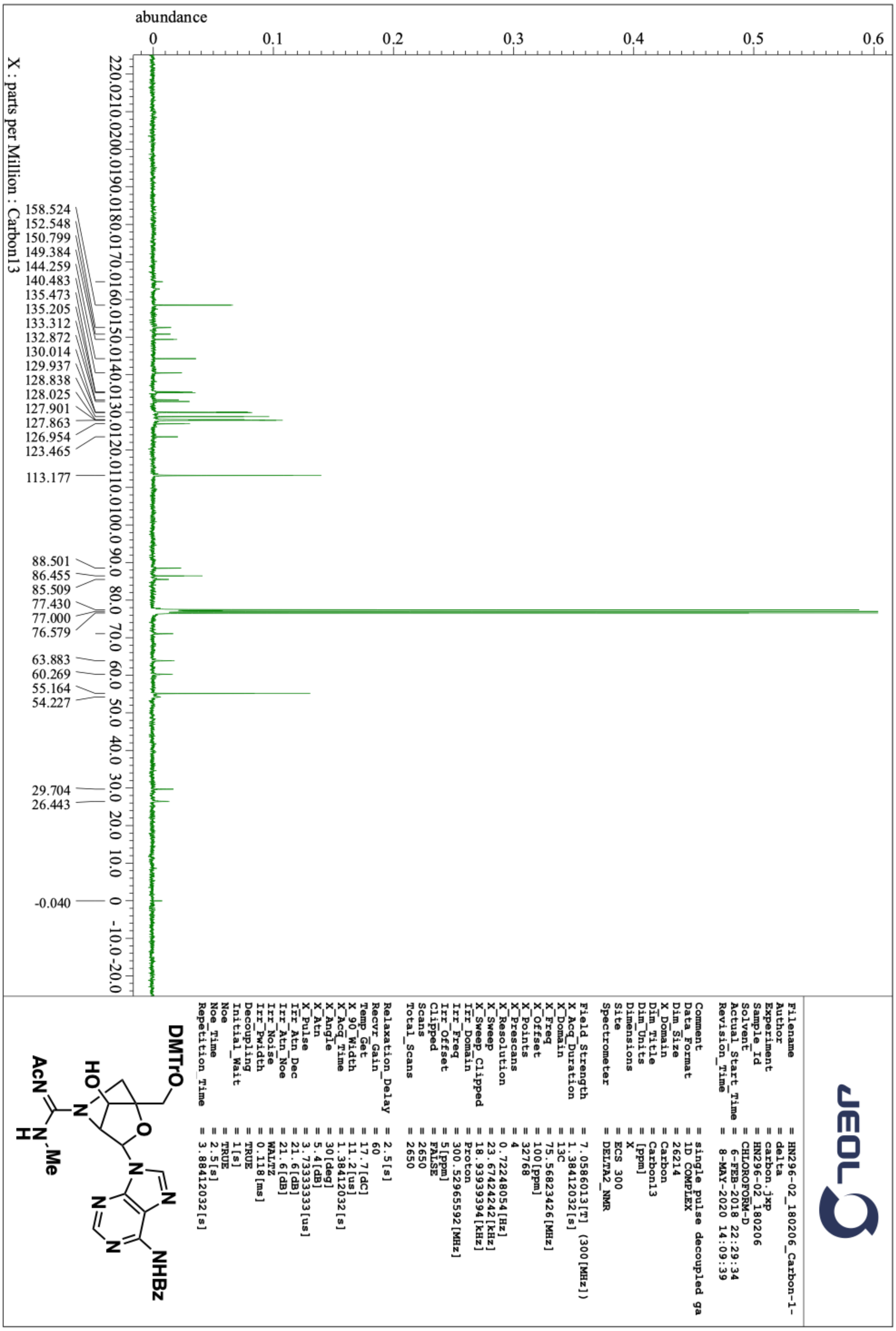

Figure S2: Compound **2a** (^{13}C NMR, $CDCl_3$, 76 MHz)

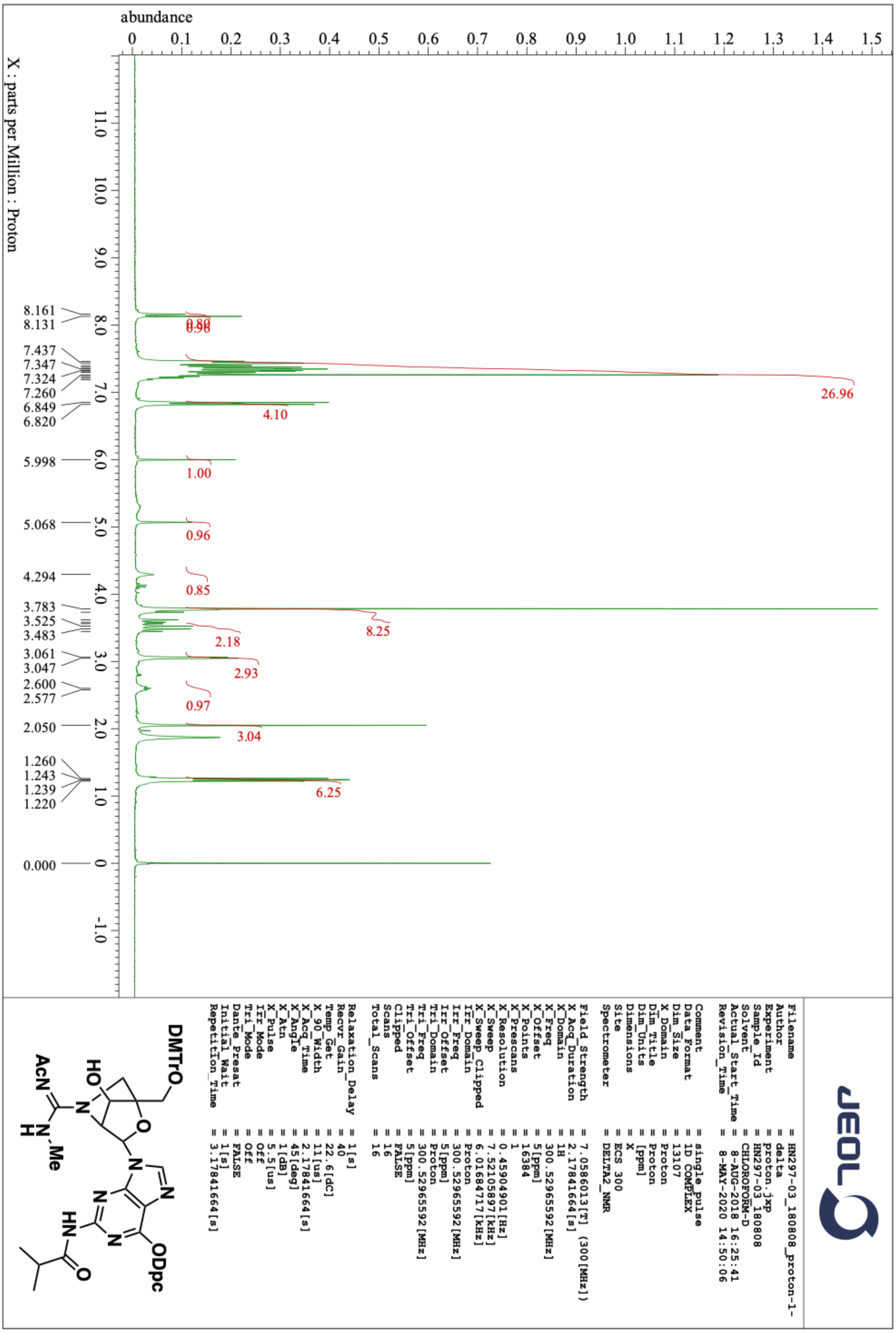

Figure S3: Compound **2b** (^{1}H NMR, $CDCl_3$, 300 MHz)

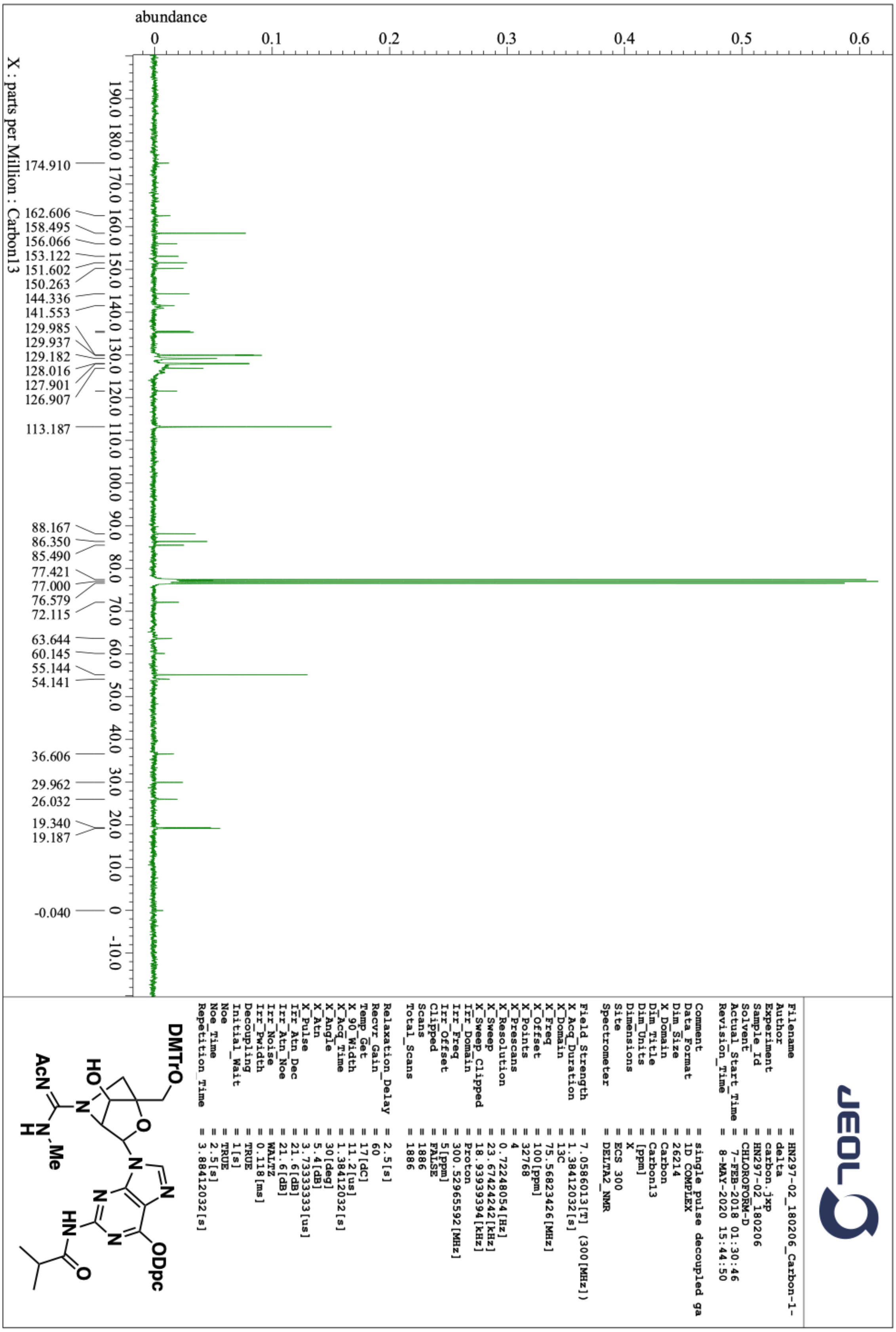

Figure S4: Compound **2b** (^{13}C NMR, $CDCl_3$, 76 MHz)

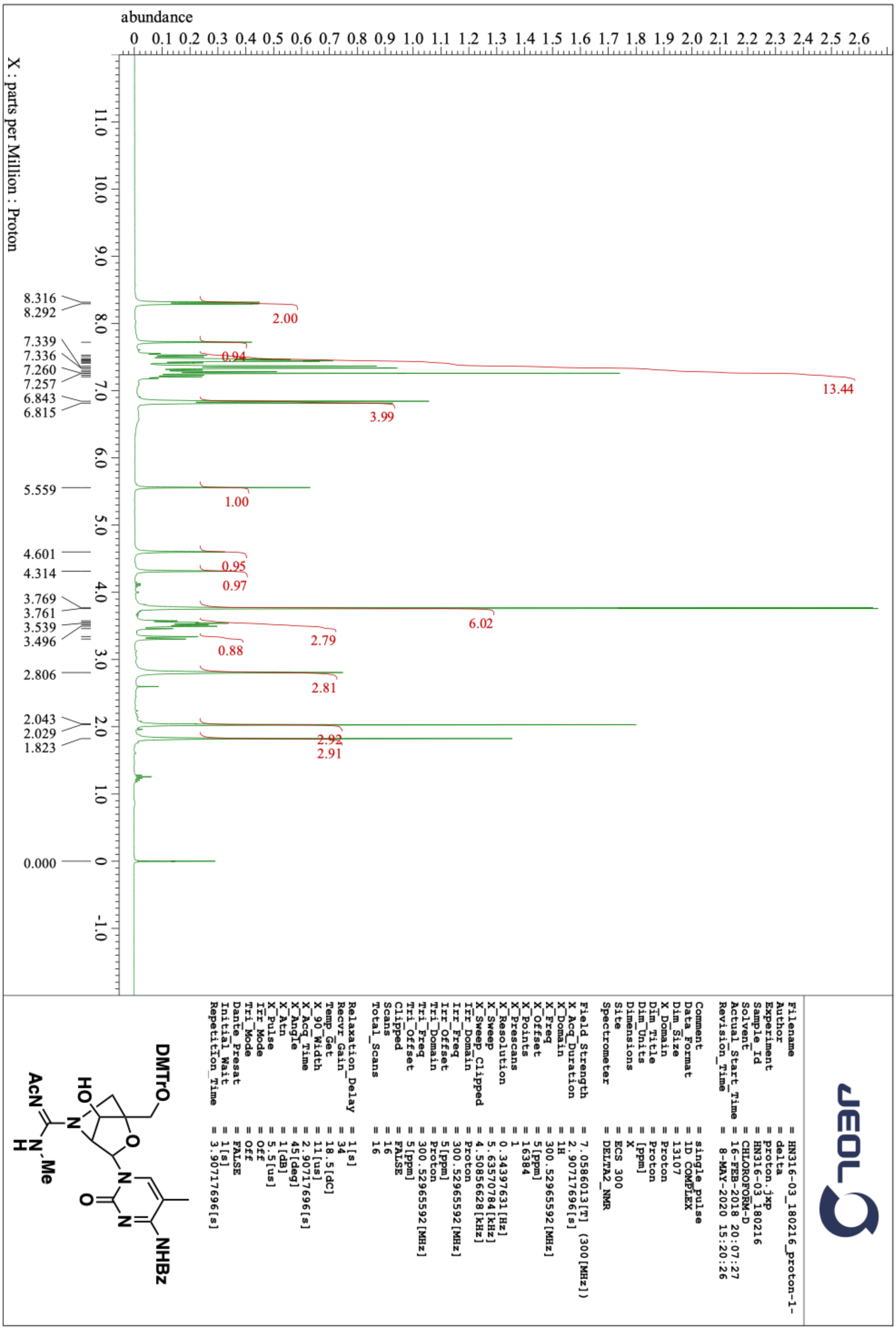

Figure S5: Compound **2c** (^{1}H NMR, $CDCl_3$, 300 MHz)

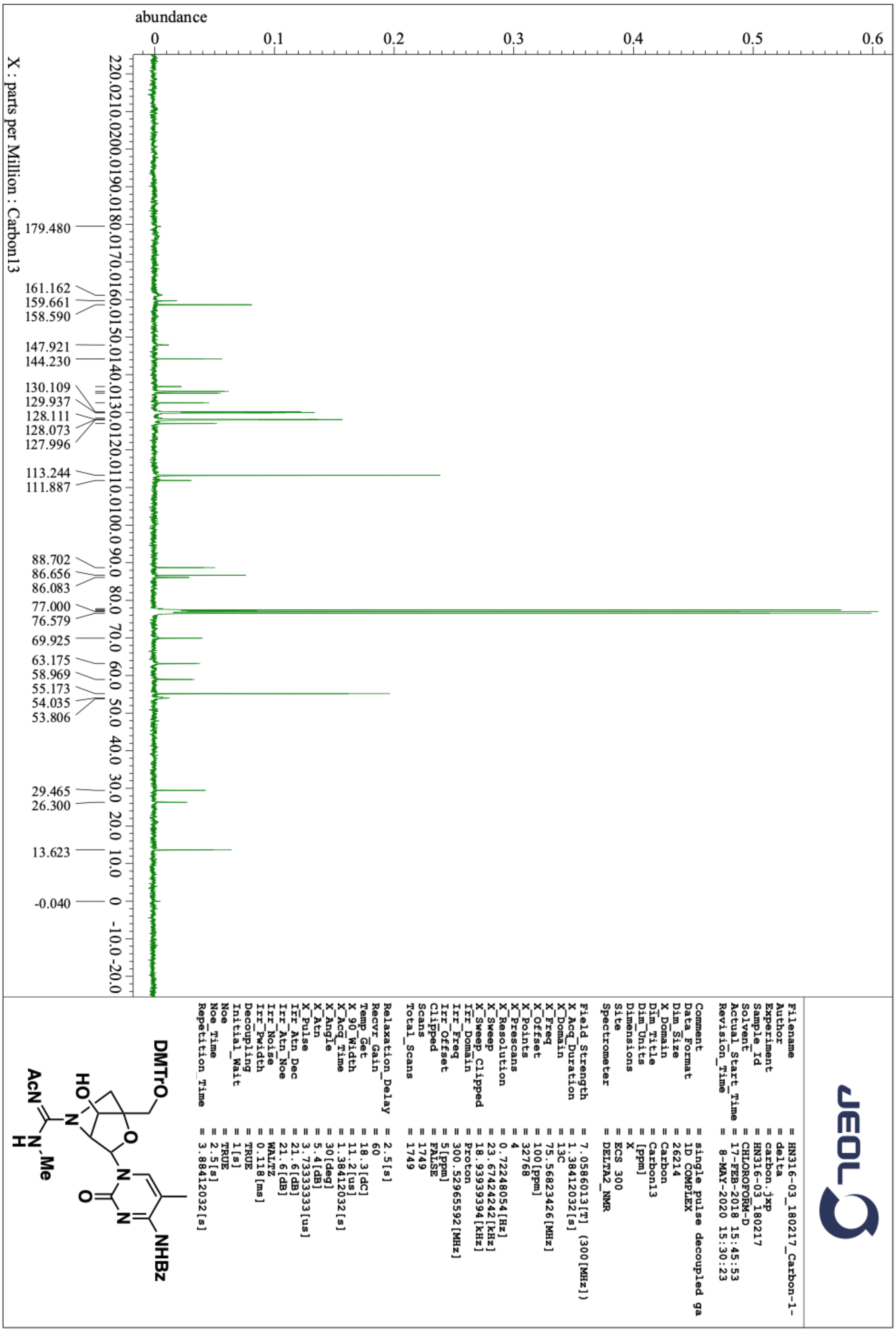

Figure S6: Compound **2c** (^{13}C NMR, $CDCl_3$, 76 MHz)

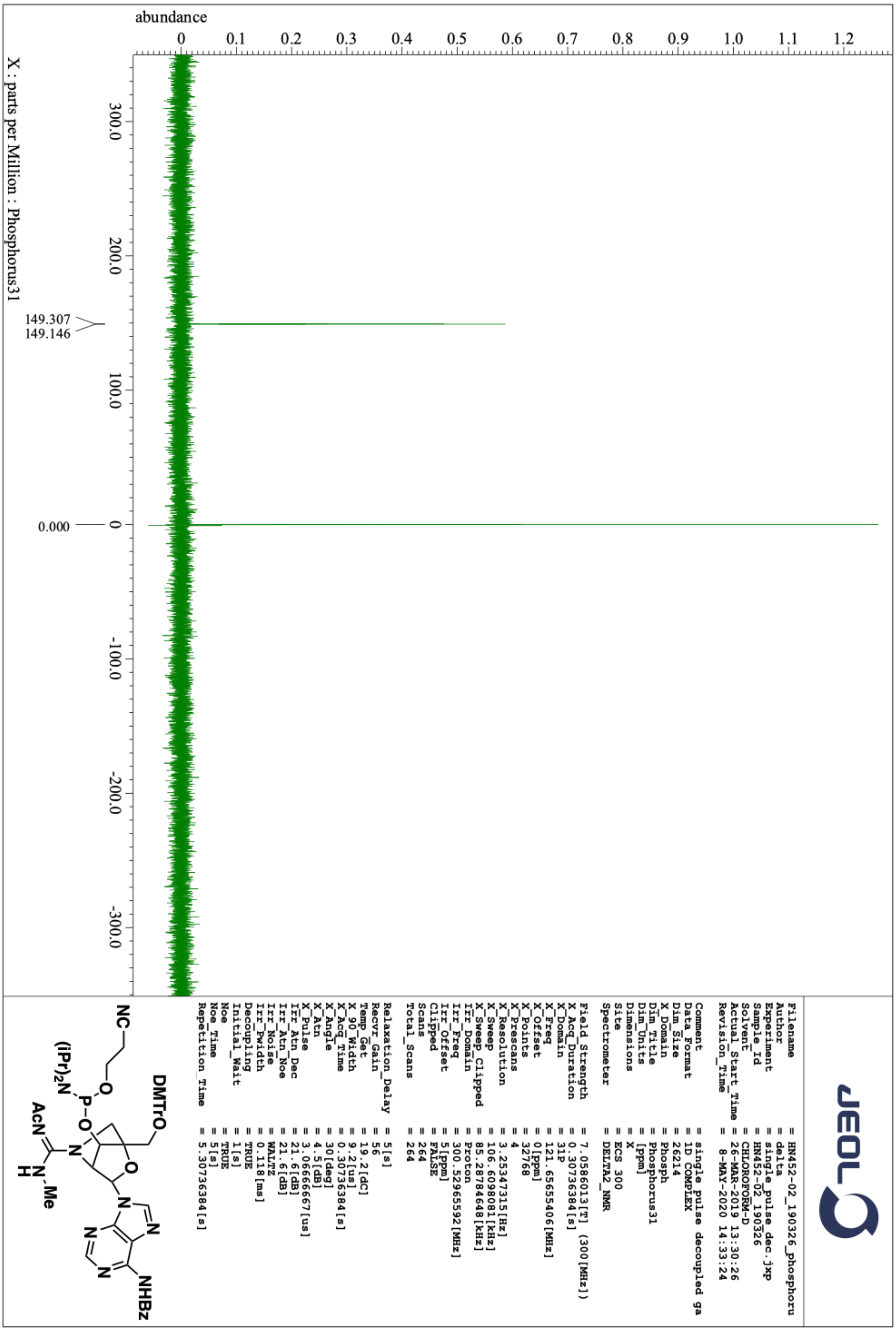

Figure S7: Compound **3a** (^{31}P NMR, $CDCl_3$, 122 MHz)

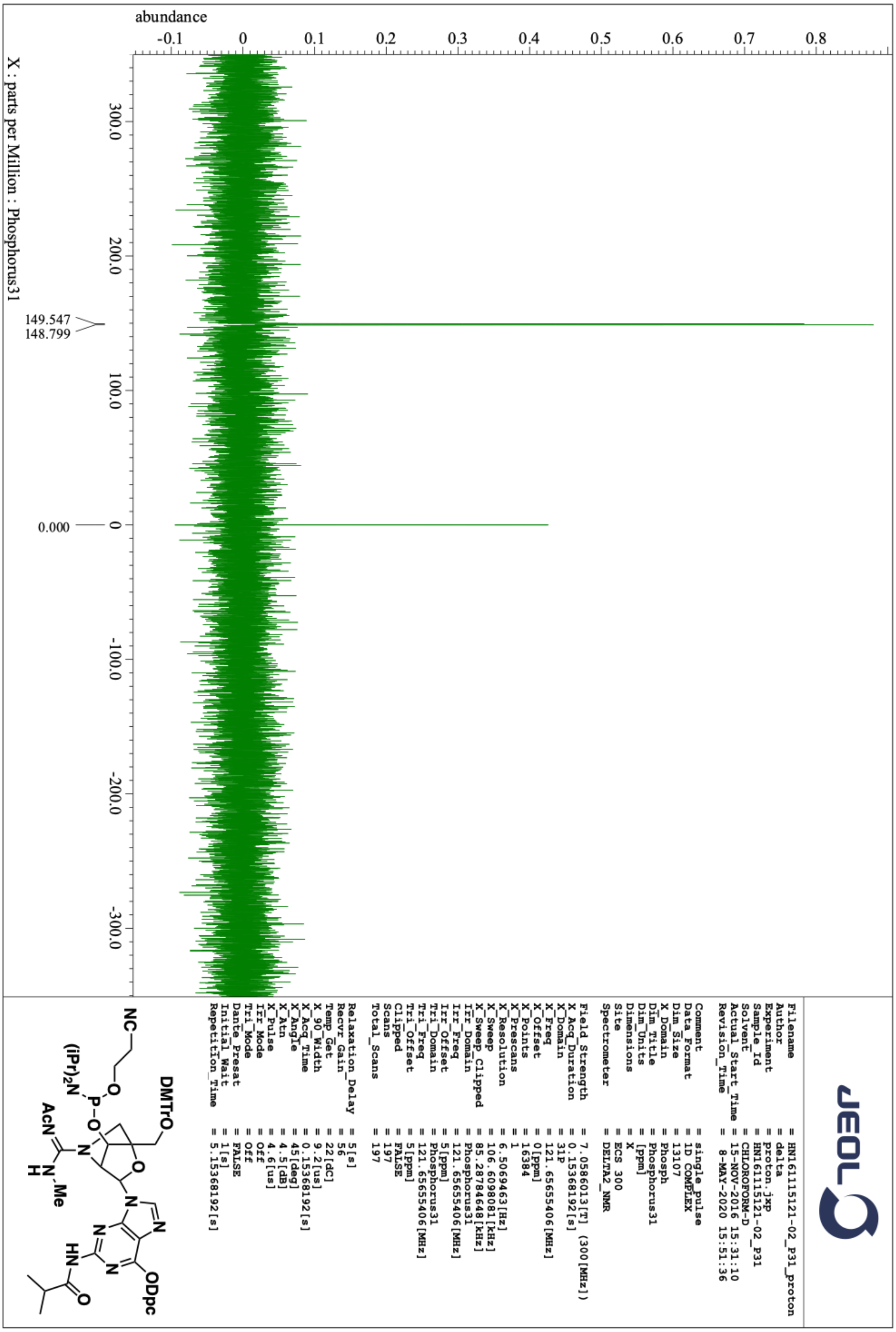

Figure S8: Compound **3b** (^{31}P NMR, $CDCl_3$, 122 MHz)

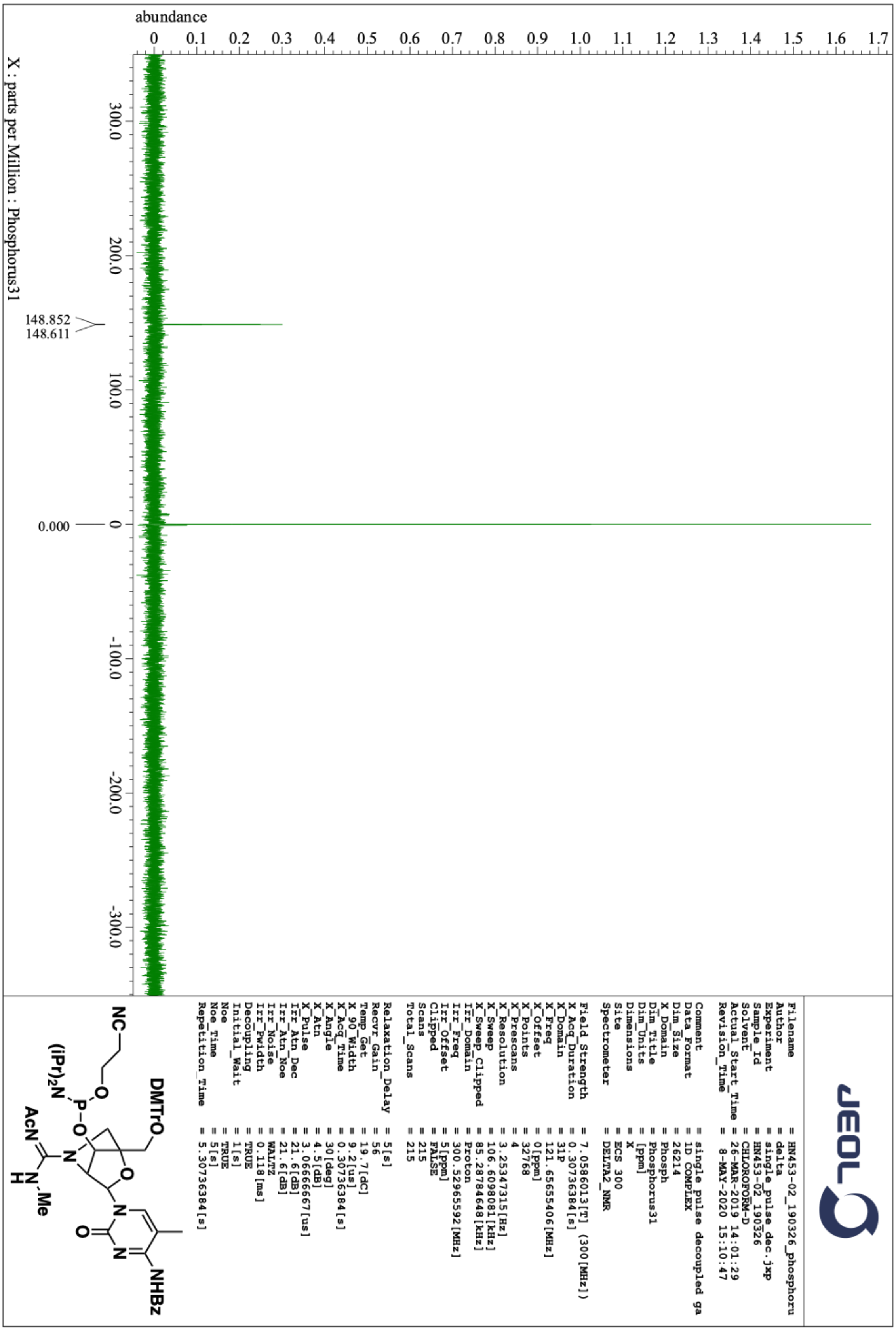

Figure S9: Compound **3c** (^{31}P NMR, $CDCl_3$, 122 MHz)

2. Characterisation of oligonucleotides

ON1 5’-d(GCG TT$\underline{A}$ TTT GCT)-3’

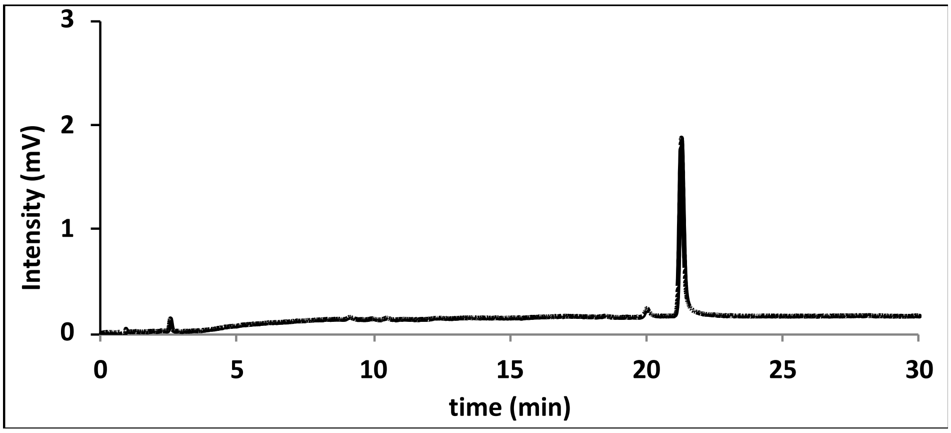

ON2 5’-d(GCG TT$\underline{G}$ TTT GCT)-3’

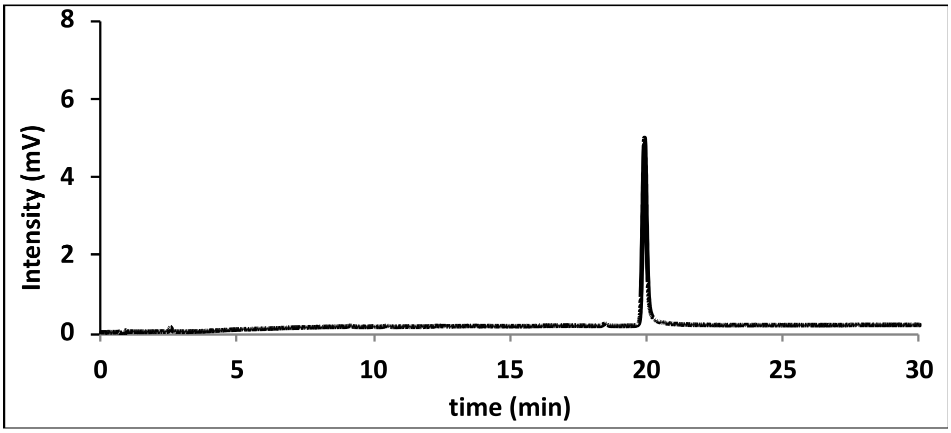

ON3 5’-d(GCG TT$\underline{^{m}C}$ TTT GCT)-3’

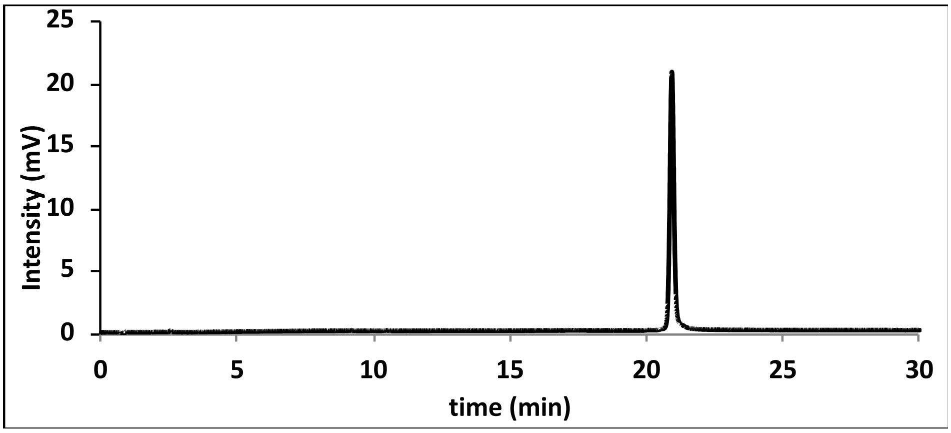

Figure S10: HPLC charts of all new oligonucleotides. HPLC conditions: Reversed-phase HPLC (Waters XBridge™ C18 column) with a linear gradient of acetonitrile (5 to 10% over 5 min, then 10 to 15% over 20 min, then 15 to 15%, over 5 min) in 0.1 M triethylammonium acetate buffer (pH 7.0). $\underline{A}$, $\underline{G}$, and $\underline{^{m}C}$ indicate GuNA[Me] modifications.

ON1 5’-d(GCG TTA TTT GCT)-3’ (A)

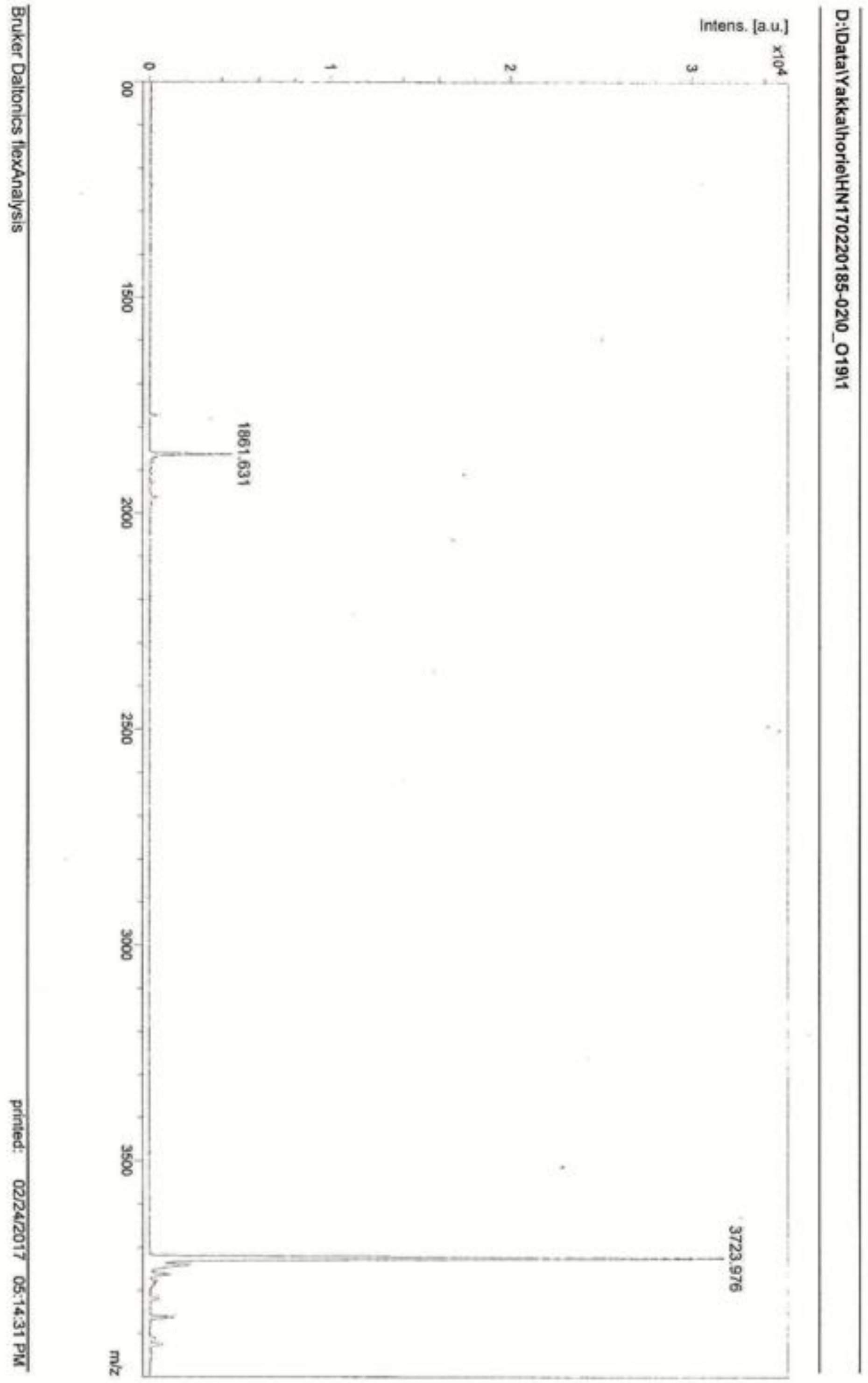

ON2 5’-(GCG TTG TTT GCT)-3’ (B)

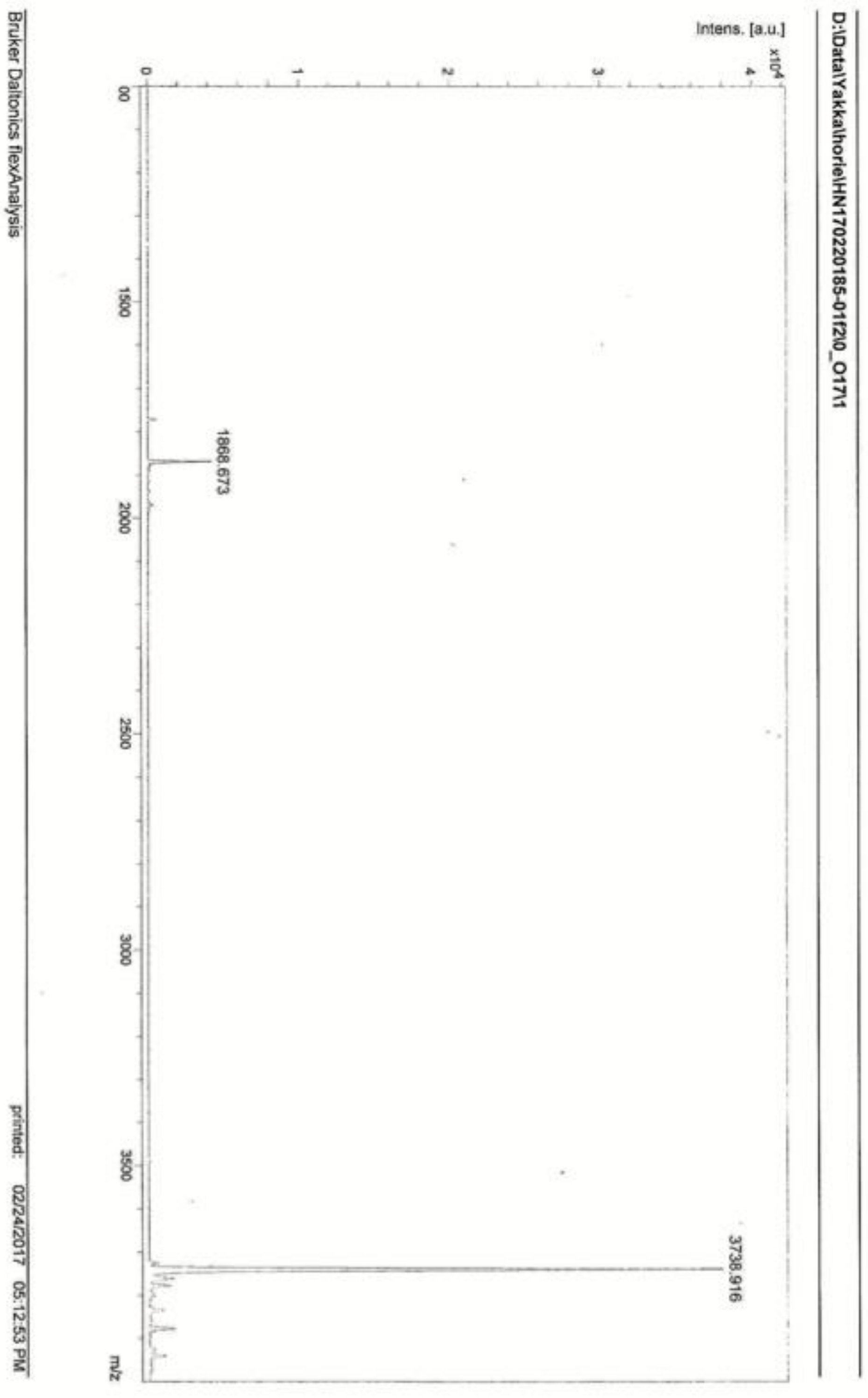

ON3 5’-d(GCG TT^{m}<u>C</u> TTT GCT)-3’ (C)

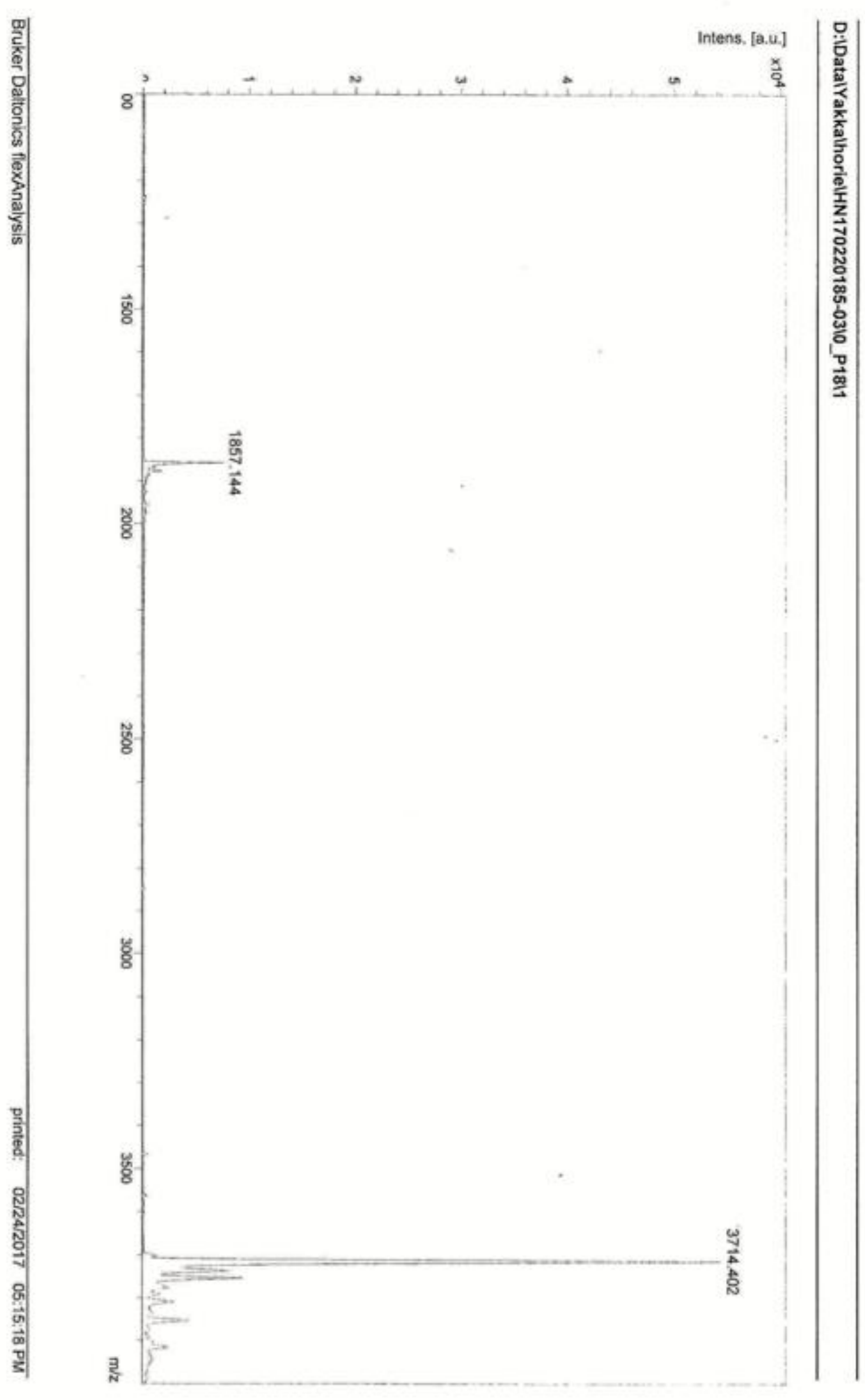

Figure S11: MALDI-TOF-MS charts of all new oligonucleotides. **ON1** 5’-d(GCG TT<u>A</u> TTT GCT)-3’ (A), **ON2** 5’-(GCG TT<u>G</u> TTT GCT)-3’ (B), **ON3** 5’-d(GCG TT^{m}<u>C</u> TTT GCT)-3’ (C); <u>A</u>, <u>G</u>, and ^{m}<u>C</u> indicate GuNA[Me] modifications.

3. UV melting experiments

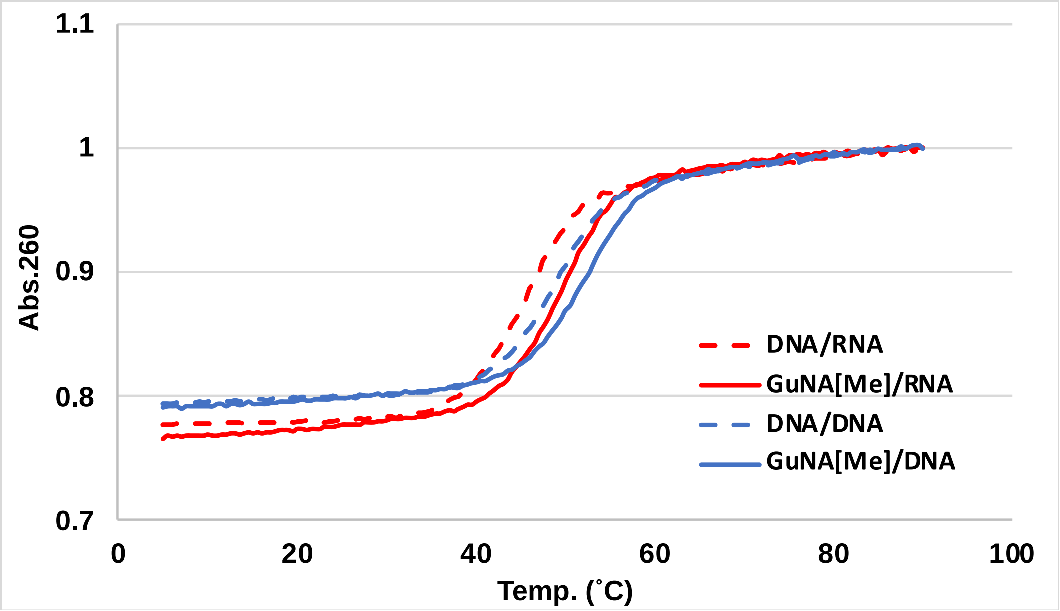

Figure S12: Normalized UV melting curves for the duplexes formed between **ON1**/**ON6** and the complementally DNA or RNA strands. The sequences are 5’-d(GCG TT<u>A</u> TTT GCT)-3’ and 5’-r/d(AGC AAA YAA CGC)-3’, respectively.

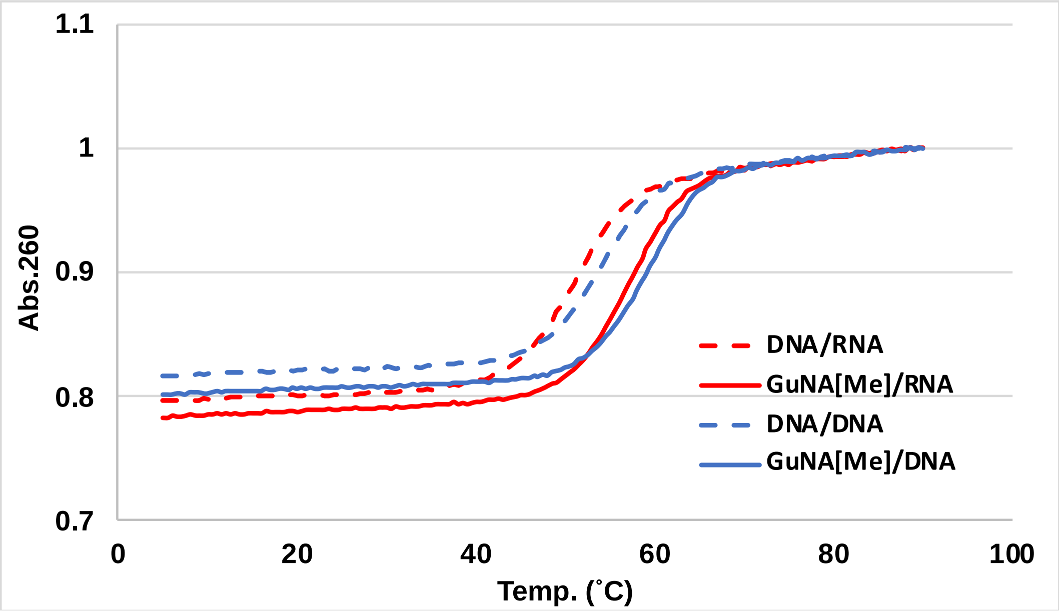

Figure S13: Normalized UV melting curves for the duplexes formed between **ON2**/**ON7** and the complementally DNA or RNA strands. The sequences are 5’-d(GCG TT<u>G</u> TTT GCT)-3’ and 5’-r/d(AGC AAA CAA CGC)-3’, respectively. Y indicates U for RNA, and T for DNA.

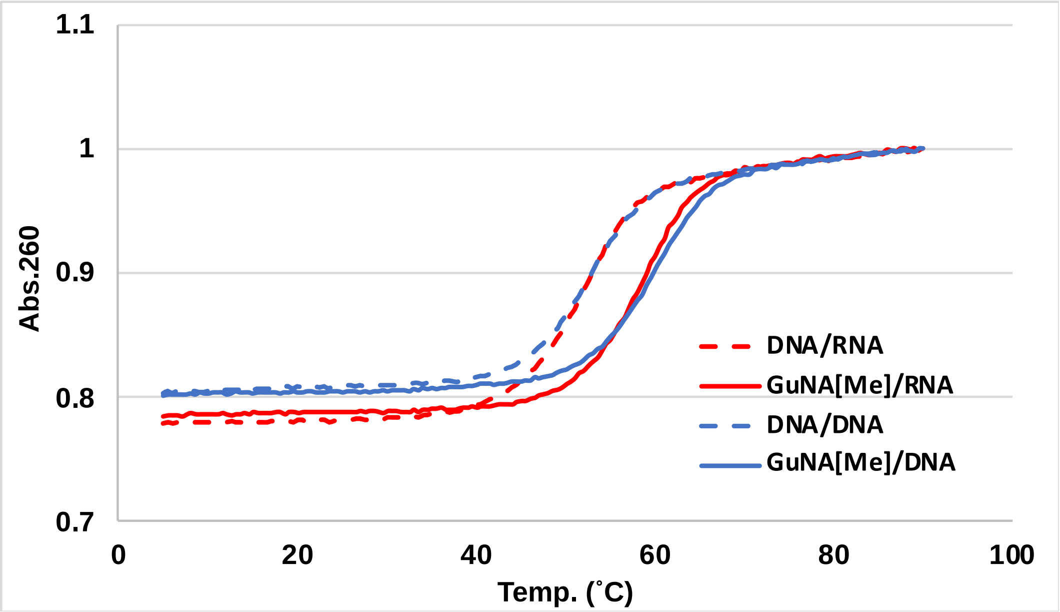

Figure S14: Normalized UV melting curves for the duplexes formed between **ON3**/**ON8** and the complementally DNA or RNA strands. The sequences are 5'-d(GCG TT^{m}C̲TTT GCT)-3' and 5'-r/d(AGC AAA GAA CGC)-3', respectively.

4. CD spectral analysis

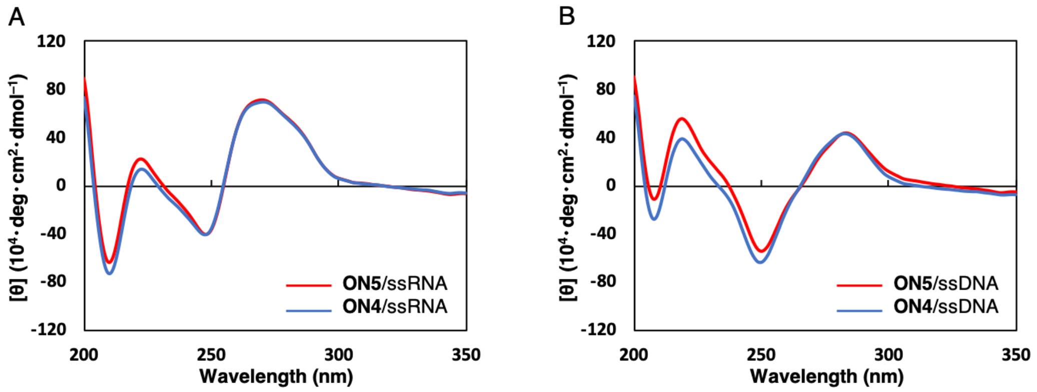

Figure S15: CD spectra of the **ON5**/ssRNA, **ON4**/ssRNA, **ON5**/ssDNA and **ON4**/ssDNA duplexes. Conditions: 10 mM sodium phosphate buffer (pH 7.2), 100 mM NaCl, 4 µM each oligonucleotide. Sequences of the complementary ssRNA and ssDNA are 5'-r(AGC AAA AAA CGC)-3' and 5'-d(AGC AAA AAA CGC)-3', respectively.
